# Supplementary material for: A Biophysical Approach to the Identification of Novel ApoE Chemical Probes
Source: Biomolecules. 2019 Jan 29;9(2):48. doi: 10.3390/biom9020048 (PMC6406525; doi:10.3390/biom9020048)
Supplement: Supplementary file 1 [file biomolecules-09-00048-s001.pdf]

Supplementary Figures

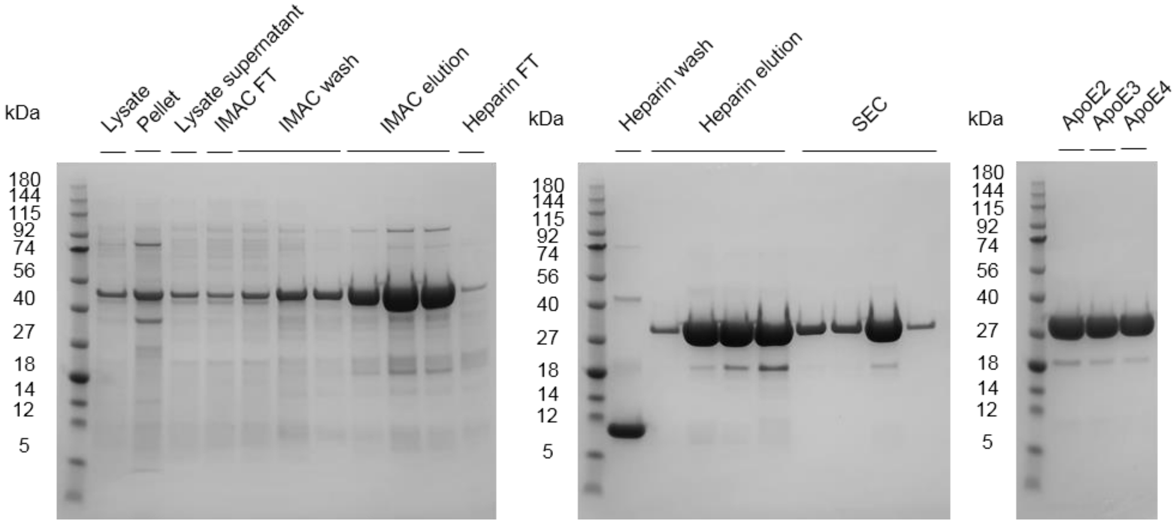

Figure S1. Summarizing gels of ApoE purification. Exemple gels of the purification of ApoE4 (left and middle), as well as a summary of final purities are shown (right). ApoE was purified by a combination of immobilized metal affinity chromatography (IMAC), heparin affinity chromatography and size exclusion chromatography (SEC). Final purification products are shown on the right with purities > 95 %. A minor impurity is found at ~22 kDa.

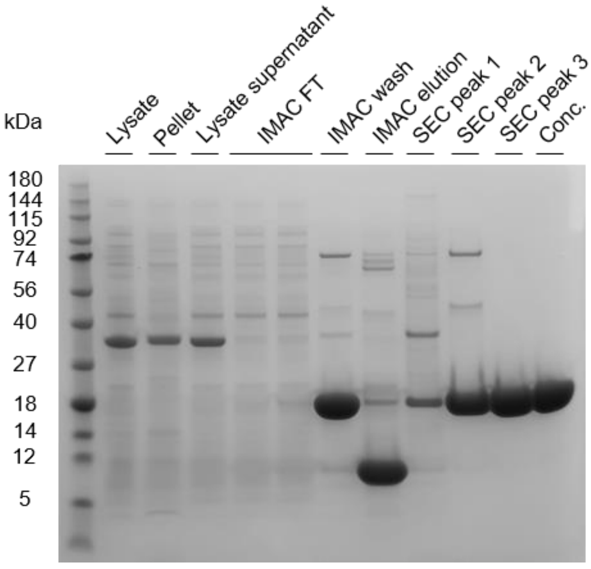

Figure S2. Purification of ApoE41-191. The ApoE4 amino terminal domain (ApoE41-191) was purified by a combination of immobilized metal affinity chromatography (IMAC) and size exclusion chromatography (SEC). Three apparent elution peaks were observed during SEC that consisted of an aggregate peak 1, higher oligomeric species peak 2 and the monomeric ApoE1-191 peak 3. Peak 3 was pooled and concentrated. Purity is > 95 %.

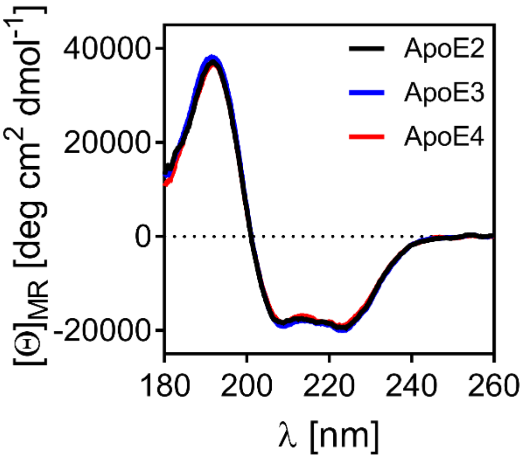

Figure S3. Circular dichroism full-length ApoE. Far UV CD spectra of ApoE isoforms (25 μM in 20 mM sodium phosphate, pH 7.4, 21°C) show correct folding and comparable α-helical content.

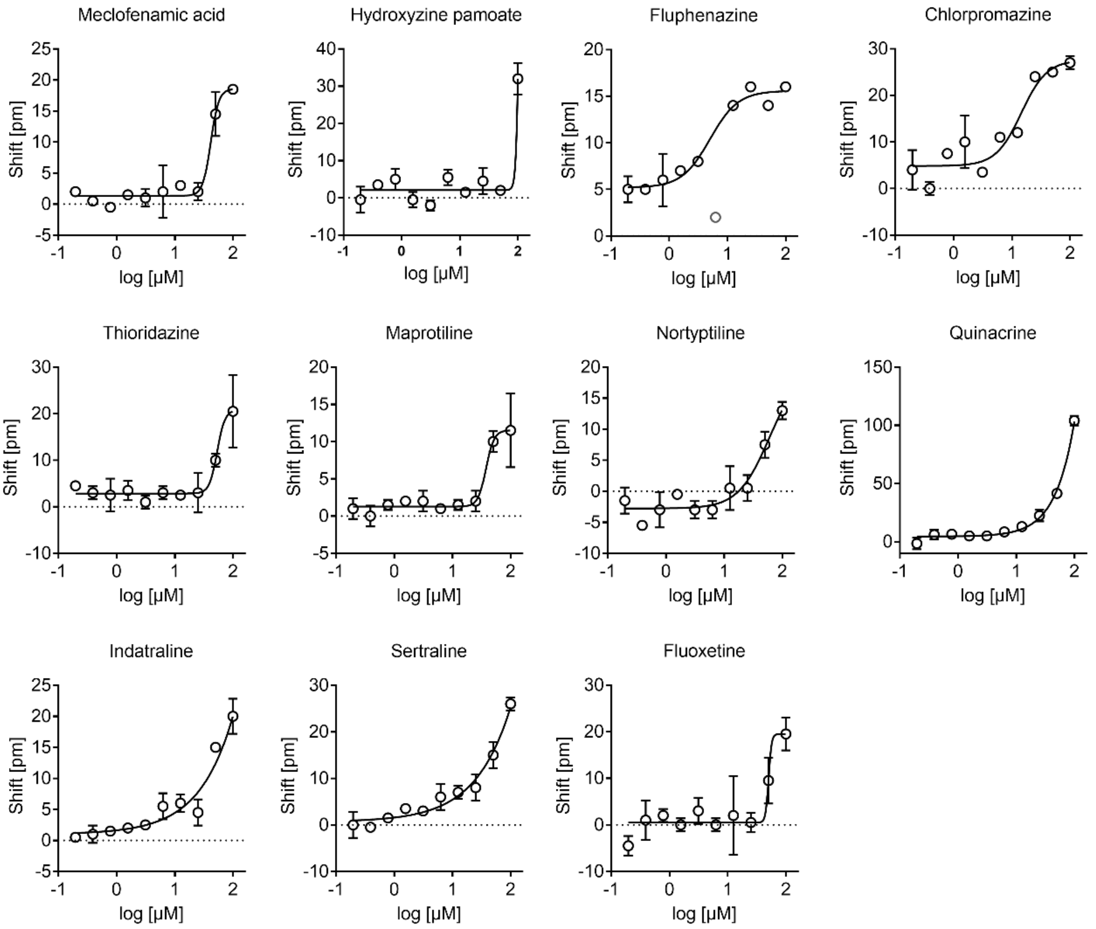

Figure S2. Confirmation of hits on the Corning Epic. Due to a production issue which limited the availability of Corning Epic plates, 45 out of the 59 compounds were prioritized based on quantitative response and chemical attractiveness and tested by concentration response on the Corning Epic. Fourteen of these hits gave full or partial binding curves that are shown here and in Figure 5C. Hits that had to be excluded were however tested by MST that identified L-Thyroxine as presented in Figure 6C.

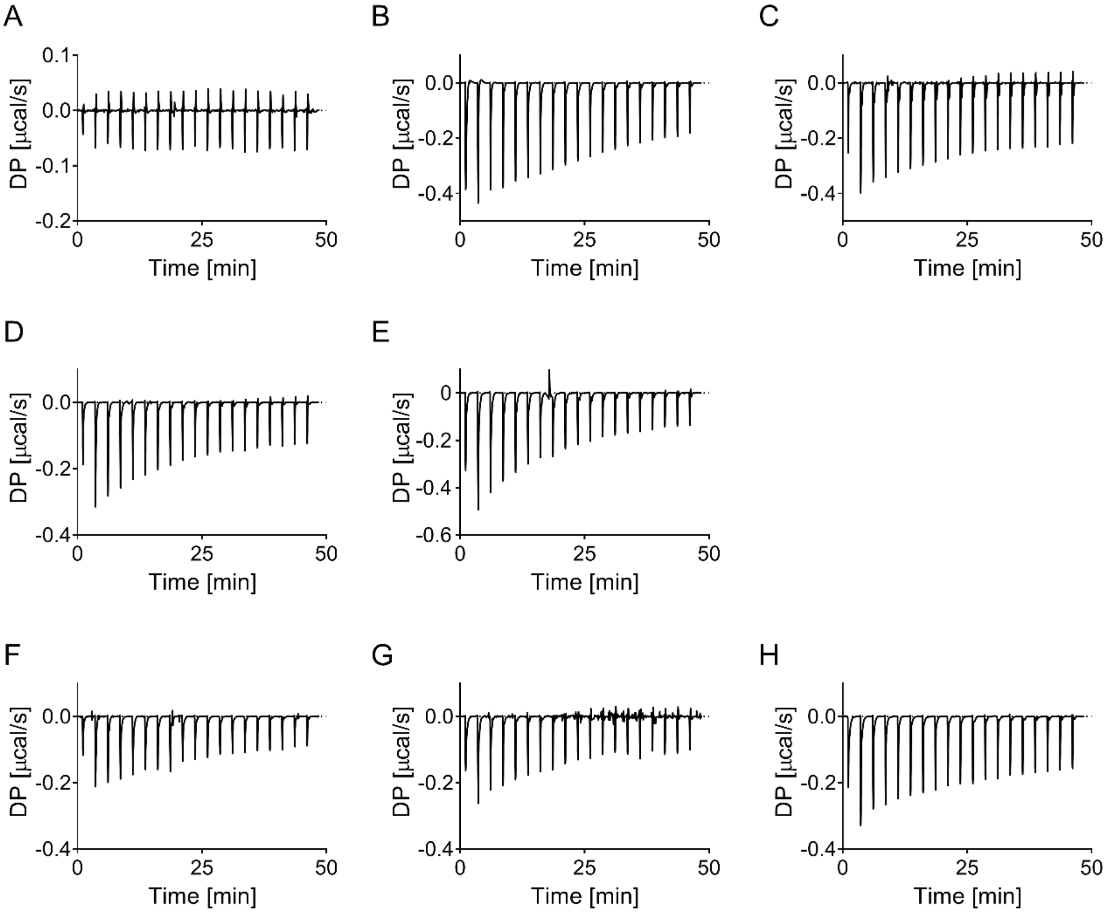

Figure S3. Isotherms of raw titration. Shown are the isotherms of raw titration heat of 300 µM ApoE4 titrated into (**A**) buffer (PBS + 0.01 % (*v/v*) Triton X-100 + 2 % (*v/v*) DMSO), (**B**) 50 µM L-Thyroxine, (**C**) 25 µM Tafamidis, (**D**) 25 µM Meclofenamic acid, (**E**) 25 µM Pamoic acid, (**F**) 35 µM Clomifene, (**G**) 50 µM Tamoxifen and (**H**) 50 µM Toremifene.

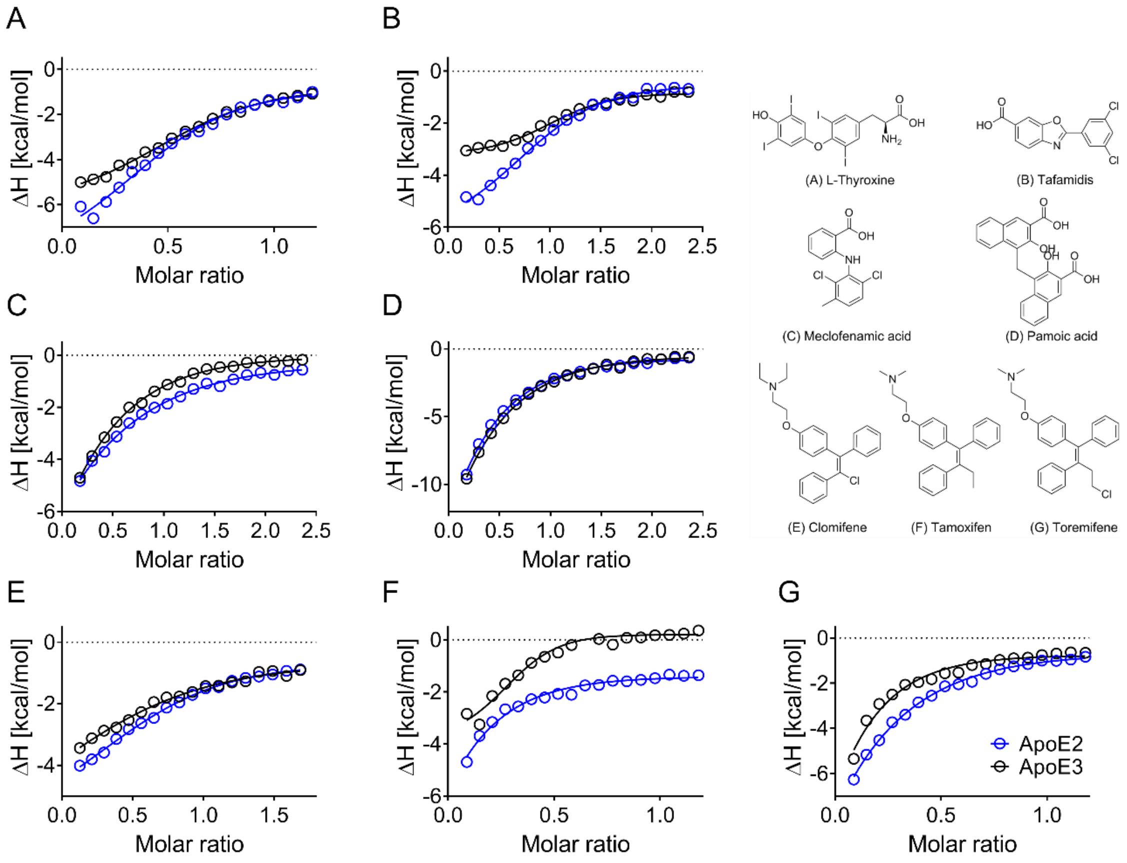

Figure S4. Hit confirmation by isothermal titration calorimetry. All confirmed hits were also tested against ApoE2 and ApoE3. Binding curves could not be fully resolved of all hits due to binding affinities being in the micromolar range. Binding to ApoE2 and ApoE3 was confirmed for (**A**) L-Thyroxine, (**B**) its compound analogue Tafamidis, (**C**) Meclofenamic acid, (**D**) Pamoic acid, (**E**) Clomifene, (**F**) Tamoxifen and (**G**) Toremifene. Shown are the normalized binding heats with the solid line representing nonlinear least square fits using single-site binding model.

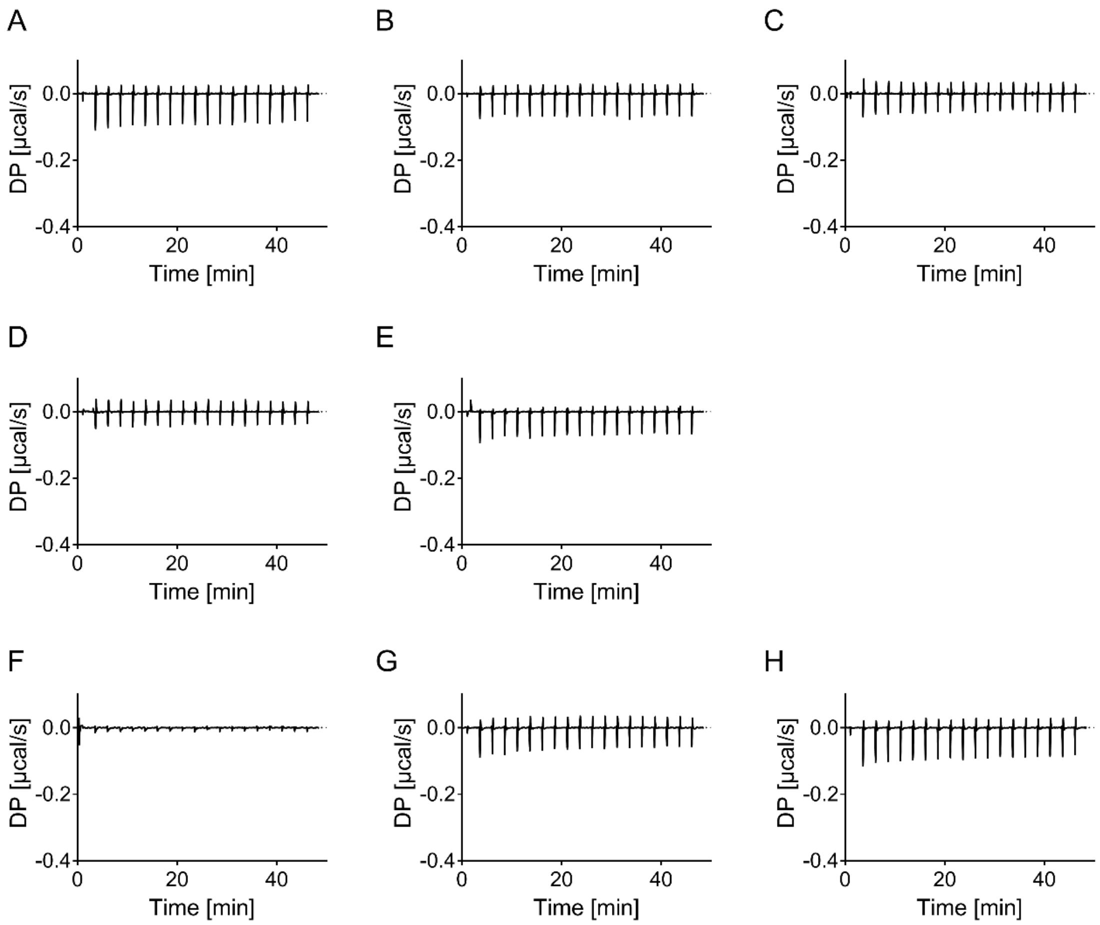

Figure S5. Isotherms of raw titration ApoE41-191. Shown are the isotherms of raw titration heat of 300 µM ApoE41-191 titrated into (**A**) PBS + 0.01 % (*v/v*) Triton X-100 + 2 % (*v/v*) DMSO, (**B**) 50 µM L-Thyroxine, (**C**) 25 µM Tafamidis, (**D**) 25 µM Meclofenamic acid, (**E**) 25 µM Pamoic acid, (**F**) 35 µM Clomifene, (**G**) 50 µM Tamoxifen and (**H**) 50 µM Toremifene.
